# A theoretical decision model to help inform advance directive discussions for patients with COPD

**DOI:** 10.1186/1472-6947-10-75

**Published:** 2010-12-20

**Authors:** Negin Hajizadeh, Kristina Crothers, R Scott Braithwaite

**Affiliations:** 1 Yale Center for Medical Informatics, Yale University School of Medicine, New Haven, USA; 2 Department of Medicine, Division of Pulmonary and Critical Care Medicine, University of Washington, Seattle, USA; 3 Section on Value and Comparative Effectiveness, Division of General Internal Medicine, New York University School of Medicine, New York, USA

## Abstract

**Background:**

Advance directives (AD) may promote preference-concordant care yet are absent in many patients with Chronic Obstructive Pulmonary Disease (COPD). In order to begin to inform AD discussions between clinicians and COPD patients, we constructed a decision tree to estimate the impact of alternative AD decisions on both quality and quantity of life (quality adjusted life years, QALYs).

**Methods:**

Two aspects of the AD were considered, *Do Not Intubate *(*DNI*; i.e., no invasive mechanical ventilation) and *Full Code *(i.e., may use invasive mechanical ventilation). Model parameters were based on published estimates. Our model follows hypothetical patients with COPD to evaluate the effect of underlying COPD severity and of hypothetical patient-specific preferences (about long-term institutionalization and complications from invasive mechanical ventilation) on the recommended AD.

**Results:**

Our theoretical model recommends endorsing the *Full Code *advance directive for patients who do not have strong preferences against having a potential complication from intubation (ETT complications) or being discharged to a long-term ECF. However, our model recommends endorsing the *DNI *advance directive for patients who do have strong preferences against having potential complications of intubation and are were willing to tradeoff substantial amounts of time alive to avoid ETT complications or permanent institutionalization. Our theoretical model also recommends endorsing the *DNI *advance directive for patients who have a higher probability of having complications from invasive ventilation (ETT).

**Conclusions:**

Our model suggests that AD decisions are sensitive to patient preferences about long-term institutionalization and potential complications of therapy, particularly in patients with severe COPD. Future work will elicit actual patient preferences about complications of invasive mechanical ventilation, and incorporate our model into a clinical decision support to be used for actual COPD patients facing AD decisions.

## Background

Advance directives (AD) allow patients to specify preferences about the care they would receive in the event of acute illness, and are recommended for comprehensive medical care [1-3]. However, compliance with AD specification is < 15% in the general population [4]. While federal policy supports AD [5], it focuses primarily on the inpatient setting. Lack of AD discussions in the outpatient setting may postpone the discussion inappropriately to the setting of acute illness, when patients may be too sick to consider their options carefully [6,7]. Indeed, only 25% of patients have AD at the time end of life decisions must be made [4] which could lead to patient dissatisfaction and misguided use of limited healthcare resources [8-10].

Barriers to discussing AD in the outpatient setting include both patient and physician discomfort; fear that the discussion will cause anxiety or take away hope; and lack of patient-tailored information [11-13]. Lack of tailored information is a particularly important barrier, as most AD use vague and unintuitive hypothetical scenarios [14,15], rather than the patient-specific information relevant to individual decision making [16]. Prognostic estimates are more accurate when based on disease-specific outcomes, and patients prefer disease-specific AD information [17].

Chronic Obstructive Pulmonary disease (COPD) is a progressive illness that exemplifies the need for AD discussions, as many patients will experience exacerbations requiring hospital admission. A decision about mechanical ventilation is an important component of AD and can prepare patients for possible treatment scenarios. While intubation and other life-saving interventions can be offered, the outcomes may not always be consistent with a patient's preferences. Decision analytic modeling can synthesize evidence based knowledge to estimate the outcomes of decisions and provide a recommended decision but has not been used before to inform the content of AD. Therefore, we constructed a theoretical decision analytic model using disease-specific information for COPD, to begin to assist COPD patients and their health care providers in the discussion of AD.

## Methods

To inform the AD discussion for COPD patients, we developed a decision model for advance directives that could accommodate a wide array of patient preferences. Decision analytic modeling is used for complex decision making in which there are competing treatments and prognoses. Treatment pathways and outcomes are represented explicitly, often using computer simulation, with probabilities based on published clinical studies. The 'preferred' or 'recommended' decision is that which maximizes the expected value of the outcome of interest, such as survival, quality of life or cost-effectiveness. Modeling is used to supplement clinical data in situations when the influential variables of the decision need to be discovered and when there is uncertainty about clinical inputs. A well-designed decision model can function as a virtual clinical trial, with the benefit of being able to change all the parameters individually or simultaneously to test the effect on outcomes and to discover the most influential variables.

We constructed our decision analytic model with two alternative decisions for the AD, *Do Not Intubate *([*DNI*] i.e., no invasive mechanical ventilation) and *Full Code *(i.e., may use invasive mechanical ventilation if necessary) in the event of respiratory failure from a COPD exacerbation. Our outcome of interest was a combination of survival and quality of life (QALYs). We focused on COPD exacerbation as the most common cause of respiratory failure requiring hospitalization in patients with COPD. We performed analyses for three scenarios of COPD severity (*mild*, *moderate *and *severe*), using GOLD criteria [18]. We then used hypothetical patient preferences about discharge location and complications of intubation to evaluate the effect on the recommended AD.

### Model overview

We constructed a decision tree using TreeAge software (Version 1.0.2, 2009, Williamstown MA) to model the impact of yearly AD decisions on quality-adjusted life-years (QALYs). QALYs are a measure of disease burden that integrates quality with quantity of life.

### Model structure

**Figure 1:**
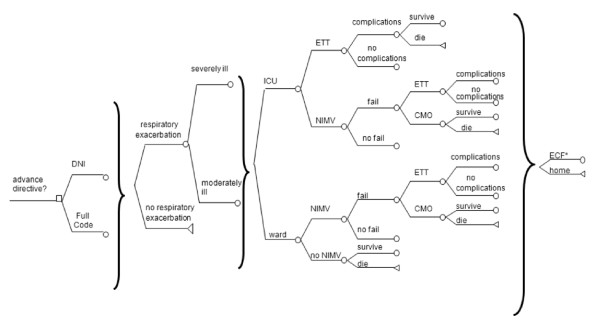
**The advance directives decision model**. The square node at the left of the diagram is a "choose" node, representing the choice of endorsing a *DNI *vs. *Full Code *AD. The circles at the origin of each branch are chance nodes, representing events that may or may not happen with a specified probability. After being admitted to the hospital with an exacerbation patients could be admitted to either the intensive care unit (ICU) or a regular ward (Ward), with non-ventilatory treatment (*no NIMV*) only offered on the Ward and ETT only in the ICU. Patients who failed mechanical ventilation could opt for no further treatment, (Comfort Measures Only; "*CMO*"). The triangles at the end of each path (the 'terminal node') represent the health effects associated with the full sequence of events in the path Paths end in death; discharge to either extended care facility for a short term or a long-term; or discharge to home. * ECF discharge is either permanent institutionalization in an ECF (*long-term ECF*), or temporary institutionalization in an ECF followed by return to home (*short-term ECF*). Discharge to long-term ECF occurred only in the pathways where there were complications of mechanical ventilation or in patients who survived CMO.

Our model follows hypothetical patients with COPD who are having annual AD discussions (Figure 1). Treatment pathways specify location of treatment (*Intensive Care Unit *[ICU] vs. *regular ward*) and intensity of treatment (*mechanical ventilation invasively with endotracheal tube *[ETT] vs. *noninvasive mechanical ventilation *[NIMV] vs. *medical treatment without mechanical ventilation *vs. *no medical interventions *[Comfort Measures Only, (CMO)]).

### Data used in the model

Three types of data are used in the model: transition probabilities (the probabilities of moving from one branch of the decision tree to the next branch), utilities (values placed on being in a given state of health), and life expectancies (Additional file 1). All data was extracted from published clinical trials when available.

#### Transition Probabilities

Probabilities used in the model specify treatment pathways (ETT vs. NIMV vs. no mechanical ventilation vs. CMO), their short term outcomes, and their long-term outcomes. Data for the probability of ETT was stratified by severity of respiratory exacerbation (*severely ill *vs. *moderately ill*) and by code status. Severe respiratory exacerbation (*severely ill*) was defined as a pH < 7.29, which was chosen because it was the prevalent threshold in the literature. We used expert opinion for the probability of mechanical ventilation for *DNI *patients as this data was not available.

"Short term outcomes" were outcomes that occurred in the hospital, and included successful weaning from mechanical ventilation, complications of ventilator support, and death. The literature defines complications heterogeneously, including the inability to discontinue mechanical ventilation [19-21] and end organ damage (e.g., sepsis from ventilator associated pneumonia, renal failure, septic shock and cardiovascular collapse) [22-24]. To reduce heterogeneity we defined ETT complications as end organ damage, infection, or the inability to discontinue mechanical ventilation. NIMV complication was defined as the inability to wean from mechanical ventilation, based on the available literature[22,23,25-33].

Long-term outcomes of treatment include permanent institutionalization in an extended care facility (*long-term ECF*), temporary institutionalization for rehabilitation followed by return to home (*short-term ECF*), or discharge to home, and were dependent on the baseline severity of COPD exacerbation and preceding short-term outcomes [21].

#### Utilities

A utility is a preference-weighted, generic, quality of life measure on a scale of 0-1. We estimated COPD utilities based on reported estimates for chronic lung diseases [34]. We calculated the utility of discharge to long-term ECF and the utility of ETT complications using time tradeoff scenarios in which hypothetical patients were asked how much time in their current state of health they would tradeoff to avoid 1 month of complications from intubation [35]. These utilities had negative values (corresponding to states worse than death) if the patient was willing to tradeoff large amounts of time alive to avoid 1 month of intubation and associated complications.

#### Life expectancy

We estimated life expectancy (LE) in COPD based on the BODE index data on COPD survival [36]. The mean age for the cohort used to determine COPD survival probabilities was 66, which was similar to the mean age of 70 for hospitalization for COPD exacerbation [37,38]. We estimated LE in a long-term ECF from a study of one year mortality in nursing homes, [39] and used the DEALE (*Declining Exponential Approximation of Life Expectancy*) [40], to convert survival probabilities to LE.

#### Evidence Synthesis

Rather than arbitrarily choosing single studies to inform parameter estimation, we used decision rules to pool relevant data: when the data were sufficiently homogeneous we pooled results using the random effects method of Der Simonian and Laird. Homogeneity was defined as having a Q-statistic of > 0.10, an I-statistic of < 25% and a p-value of < 0.05 with no significant outliers on Forest plot. If data were insufficiently homogeneous we used the median value as our point estimate and specified plausible ranges based on the lowest and highest reported confidence intervals. If insufficient data was available we used expert opinion and employed a wide plausible range for sensitivity analyses. Finally, back calculation was used for some variables using other parameter estimates in the decision tree.

### Sensitivity Analyses

One-way sensitivity analysis varies each variable independently across a plausible range of values (usually the 95% CI) while keeping all other variables constant to assess the influence of data uncertainty on the robustness of the model. Model robustness was determined by whether the recommended AD changed as the parameter estimates were varied across their plausible ranges, and whether the difference in QALYs between *Full Code *and *DNI *changed (eg., whether the difference in QALYs for *DNI *vs. *Full Code *changed when the lower bound of the 95% CI was used for probability of ETT complication). For the utility of long-term ECF and of complications from intubation (*ETT complications*) we used the utilities generated from the hypothetical time tradeoff scenarios.

## Results

The recommended AD decision varied substantially with hypothetical patient preferences. When hypothetical patients were not willing to tradeoff any time alive to avoid complications of intubation or long-term institutionalization, a *Full Code *AD resulted in greater QALYs than *DNI*. As patients were willing to tradeoff more time alive to avoid complications of intubation or long-term institutionalization, *DNI *became the recommended choice, particularly for patients with severe COPD.

### Hypothetical patients not willing to tradeoff time alive to avoid intubation

For hypothetical patients who did not have a strong preference against complications of intubation (i.e., were not willing to give up life expectancy to avoid complications of intubation), *Full Code *was recommended when compared to *DNI *regardless of COPD severity. However, the strength of the recommendation to be *Full Code *decreased as the severity of baseline COPD increased: for patients with mild COPD the increase in QALYs for choosing *Full Code *instead of *DNI *was 0.74 QALYs, whereas for patients with severe COPD the increase in QALYs for choosing *Full Code *instead of *DNI *was 0.13 QALYs.

### Hypothetical patients willing to tradeoff time alive to avoid intubation

**Figure 2:**
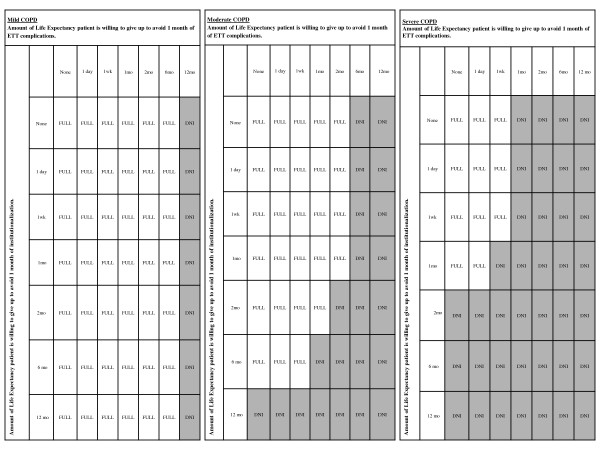
**Sensitivity Analyses of the utility of discharge to long-term ECF and of the utility of having a complication from intubation**. Results of two way sensitivity analyses are illustrated as tables with increasing willingness to tradeoff time from life expectancy (LE) to avoid discharge to long-term ECF; and to avoid having complications from intubation. The shaded regions are utilities for which the recommended directive is *DNI*. Utilities have negative values (corresponding to states worse than death) if the patient is willing to tradeoff large amounts of time alive to avoid complications from intubation. The numbers in brackets represent the calculated utilities. Three separate figures correspond to the effect of preferences on the AD decision for different severities of baseline COPD. For patients with mild COPD (Figure 3a), *DNI *becomes the recommended directive only when the patient is willing to tradeoff more than 1 year of LE to avoid complications of intubation. For patients with moderate COPD (Figure 3c), *DNI *becomes the recommended directive when the patient is willing to tradeoff more than 6 months of LE to avoid complications of intubation. *DNI *also becomes the recommended directive when the patient is willing to tradeoff more than 1 year of LE to avoid long-term ECF. For patients with severe COPD (Figure 3c), *DNI *becomes the recommended directive when the patient is willing to tradeoff more than 1 month of LE to avoid complications of intubation. *DNI *also becomes the recommended directive when the patient is willing to tradeoff more than 2 months of LE to avoid long-term ECF. When taking both patient preferences into account, if the patient is willing to tradeoff more than 1 week of LE to avoid complications of intubation and discharge to long-term ECF, *DNI *becomes the recommended directive.

For hypothetical patients who had a strong preference against complications of intubation *DNI *was recommended compared to *Full Code*, particularly as COPD severity increased. For patients with mild COPD, *DNI *became the recommended directive when a patient was willing to trade off ≥ 1 year to avoid 1 month of complications of intubation (Figure 2A). For patients with severe COPD, *DNI *was always the recommended AD, unless a patient was only willing to tradeoff <3 weeks of time alive to avoid 1 month of complications of intubation and/or willing to tradeoff < 2 months of life expectancy in order to avoid long-term institutionalization (Figure 2C).

### Sensitivity Analyses

**Figure 3:**
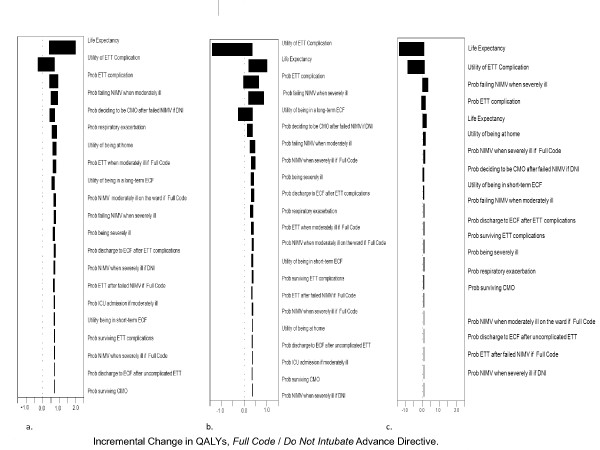
**Tornado Diagrams**. Three separate graphs correspond to the three alternative scenarios for COPD severity in our base case analyses. (a., Mild COPD; b., Moderate COPD; c., Severe COPD). Results of one way sensitivity analyses are illustrated as tornado diagrams with the horizontal bars representing the incremental change in QALYs for *Full Code *compared to *DNI *advance directive. The widest bars represent the variables that the model is most sensitive to because changes in their parameter estimates result in large changes in QALY. Variables that cross the 0 mark indicate a change in the recommended AD from *Full Code *to *DNI*. For the mild COPD scenario (Figure 2a), there is no change in the recommended directive when parameter estimates for the model variables are changed. For the moderate COPD scenario (Figure 2b), *DNI *becomes the recommended directive when the probability of having a complication from ETT increases. For the severe COPD scenario (Figure 2c), *DNI *becomes the recommended directive when the probability of having a complication from ETT increases; and when the probability of failing NIMV decreases. ETT = Invasive mechanical ventilation via endotracheal intubation; NIMV = Noninvasive mechanical ventilation; ECF = Extended Care Facility; CMO = *Comfort Measures Only*; DNI = *Do Not Intubate*; ICU = Intensive Care Unit.

We varied each input to the model across its plausible range to determine whether our results were robust (i.e., whether the recommended AD changed to *Full Code *and whether the difference in QALY changed substantially), (Figures 3A-C). We first limited these analyses to patients who were unwilling to trade off any time alive to avoid intubation or long-term institutionalization. The mild COPD scenario (Figure 3A) yielded the most robust inferences for decision making. All except one of the probability ranges included 0, indicating that plausible range variation rarely changed the recommended AD. The severe COPD scenario yielded the least robust inferences for decision making Variables that led to *DNI *being favored were an increase in the probability of ETT complications (≥ 0.617, *DNI *favored), and a decrease in the probability of failing NIMV when severely ill (i.e., higher likelihood of survival with just NIMV treatment; ≤ 0.14 *DNI *favored).

## Discussion

In this study, we constructed a theoretical decision analytic model of advance directive choices for COPD patients in the event of a COPD exacerbation. We looked at the effect of disease severity and hypothetical patient preferences on quality adjusted life years and thus the model's recommended advance directive. The variables with greatest influence on quality adjusted life years were patient preferences regarding permanent institutionalization and ETT complications as well as patients' severity of COPD. Patient preferences were most influential in patients with severe COPD: when the utility of long-term ECF was ≤ 0 (i.e., "I think living in a nursing home permanently is the same as or worse than being dead"), the recommended directive became *DNI*. Other influential variables were the probabilities of ETT complication and NIMV complication. The recommended directive also changed to *DNI *when the probability of ETT complications increased, and when the probability of NIMV failure decreased (i.e., higher likelihood of survival with just NIMV treatment).

We chose COPD-related respiratory failure in order to focus on a specific and common scenario requiring decision making. Using our results a clinician can compare and contrast prognoses with different AD choices. It is our hope that this will facilitate clinicians to initiate AD discussion with their COPD patients, incorporating their individual preferences (e.g., about long-term institutionalization). Other patient-specific factors, such as physical and psychiatric comorbidities, prior mechanical ventilation outcomes, prior admissions, baseline functional status (ADLs) and home support, may influence the probability of complications and change the recommended AD decision for individual patients, and future clinical research should explore their relative importance and their feasibility for incorporation into decision supports. Future research may also explore further developing tools to elicit the patient preferences identified by our model.

Although there was insufficient data to inform estimates for some variables requiring us to rely on a single study or on expert opinion, the influential variables on sensitivity analysis were *not *derived by expert opinion. The probability of ETT complications, however, was an influential variable for which only one study was available [41], because most studies do not distinguish between mortality from ETT and complications from ETT that lead to mortality [41]. We have thus identified an important variable to focus future clinical research in the intensive care unit. Increased data on the probability of ETT complications will improve advance directive decision making by allowing quality of life to be discussed in the event of survival after intubation. In addition, the preference-specific variables (e.g., willingness to trade off time alive to avoid intubation), were not derived from the literature. We argue that these variables are more informative if patient-specific rather than based on cohort studies from the literature. Actual patient-specific preferences will be obtained in the future by coupling the model to a decision aid that elicits patient-preferences (e.g., preferences about health states) and will allow for individually tailored advance directive recommendations.

Another important limitation of our model is that it does not use state transitions, and therefore is not able to assess the influence of multiple respiratory exacerbations within one year. Patients who have multiple exacerbations have increasing severity exacerbations and poorer outcomes than is reflected in the model [42,43]. Additionally, we assumed that the utility of discharge home after a COPD exacerbation, and LE, was the same as the utility and LE before COPD exacerbation. The literature suggests that some patients who are discharged home do not return to normal quality of life immediately, and that health related quality of life suffers for some time after the acute symptoms have resolved [43,44]. Future work includes evolving the decision tree into a Markov state transition model that can represent the clinical course of severe COPD with greater fidelity; and incorporating the model into a decision aid using patient preference to support shared decision making. Future work may also include gathering more knowledge about a wide variety of important domains, such as the effect of clinician's specialty on the AD decisions, the influence of patient-specific factors such as gender, religion, cultural background, surrogate involvement and living situation (i.e., what resources the patient has to assist with home care); and the patient's reactions to the model.

Although we believe that informing all COPD patients about alternate treatment options in the event of severe respiratory exacerbations, the ideal timing of this discussion needs to be established (e.g., after deterioration in PFTs are noted in a patient with severe COPD). Appropriate psychiatric counseling may also need to be made available in the event of any distress caused by the discussion of end of life scenarios, and future work on a decision aid will assess patient's reactions to this discussion.

## Conclusions

In summary, our model estimates both the survival from alternate advance directives as well as the resulting quality of life based on hypothetical individual patient preferences. We believe that making our model available to clinicians in the form of a decision aid, coupled with actual patient preference elicitation, will better inform AD shared decision making and is one step towards increasing preference-congruent care at the end of life.

## Competing interests

The authors declare that they have no competing interests.

## Authors' contributions

*NH *contributed to the study concept and design, the analysis and interpretation of data, and the drafting of the manuscript and critical revision for important intellectual content. NH had full access to all of the data in the study and takes responsibility for the integrity of the data and the accuracy of the data analysis. *RSB *contributed to the study concept and design, the analysis and interpretation of data, and the drafting of the manuscript and critical revision for important intellectual content. *KC *contributed to the study concept and design, the analysis and interpretation of data, and the drafting of the manuscript and critical revision for important intellectual content. All authors read and approved the final manuscript.

## Pre-publication history

The pre-publication history for this paper can be accessed here:

http://www.biomedcentral.com/1472-6947/10/75/prepub

## Supplementary Material

Additional file 1 **Table of parameter estimates and data sources**. Click here for file
