# Supplementary material for: A theoretical decision model to help inform advance directive discussions for patients with COPD
Source: BMC Med Inform Decis Mak. 2010 Dec 20;10:75. doi: 10.1186/1472-6947-10-75 (PMC3020153; doi:10.1186/1472-6947-10-75)
Supplement: Additional file 1 — Table of parameter estimates and data sources. [file 1472-6947-10-75-S1.DOCX]

| **Variable Name** | **Estimate** | | **Plausible Range** | **Method used for estimation** | | **References** |
| --- | --- | --- | --- | --- | --- | --- |
| **Probabilities** |  | | | | | |
| Respiratory Exacerbation | Mild COPD:  0.51 | | 0.40-0.60  (0.10 - 1.00)* | Published estimates  (Median, Range) and expert opinion | | [1-3] |
|  | Moderate COPD:  0.95 | | 0.70-1.00 (0.10 - 1.00)* |  |  |  |
|  | Severe COPD:  0.95 | | 0.80-1.00  (0.10 - 1.00)* |  |  |  |
| Severe respiratory exacerbation (Severely ill) | Mild COPD:  0.02 | | 0.01-0.10  (0.00 - 1.00)* | Single Study  (95%CI) | | [1, 4] |
|  | Moderate COPD:  0.19 | | 0.10-0.30  (0.00 - 1.00)* |  |  |  |
|  | Severe COPD:  0.42 | | 0.30-0.60  (0.00 - 1.00)* |  |  |  |
| ICU admission if severely ill | 1.00 | | 0.80 - 1.00 | Single study (95%CI) | | [5] |
| ICU admission if moderately ill | 0.20 | | 0.10 - 0.30 | Expert opinion | |  |
| ETT if *Full Code* when severely ill | 0.55 | | 0.02 - 0.92 | Published estimates (Median, Range) | | [6-11] |
| ETT if *DNI* when severely ill | 0.05 | | 0.01 - 0.20 | Expert Opinion (Median, Range) | |  |
| ETT if *Full Code* when moderately ill | 0.21 | | 0.06 - 0.36 | Published estimates (Median, Range) | | [6, 12] |
| ETT if *DNI* when moderately ill | 0.01 | | 0.00 - 0.02 | Expert Opinion | |  |
| NIMV when severely ill if *Full Code* | 0.16 | | 0.00 - 0.54 | Published estimates (Median, Range) | | [5, 5, 9, 13, 14] |
| NIMV when severely ill if *DNI* | 0.90 | | 0.80 - 1.00 | Expert opinion | |  |
| NIMV when moderately ill if *Full Code* on Ward | 0.72 | | 0.64 - 0.82 | Published estimates (Median, Range) | | [15, 16] |
| NIMV when moderately ill if *DNI* on Ward | 0.72 | | 0.64 - 0.82 | Expert Opinion | |  |
| Failing NIMV when severely ill | 0.28 | | 0.12 - 0.73 | Published estimates (Median, Range) | | [7, 15, 17-22] |
| Failing NIMV when moderately ill | 0.14 | | 0.10 – 1.00 | Random Effects Pooling (5 studies) (95% CI) | | [23-27] |
| ETT when failed NIMV if *Full Code* | 1.00 | | 0.90 – 1.00 | Random Effects Pooling (11 studies) (95% CI) | | [7, 8, 17, 19, 20, 24, 25, 28-30][31-33] |
| ETT when failed NIMV if *DNI* | 0.05 | | 0.00 - 0.10 | Expert opinion | |  |
| Complicated ETT (failure to liberate from ventilator, end organ dysfunction) | 0.42 | | 0.19 - 0.77 (0.00 - 1.00)* | Published estimates (Median, Range) | | [17, 18, 34] |
| Surviving complicated ETT | 0.62 | | 0.49 - 0.75 (0.00 - 1.00)* | Random Effects Pooling (2 studies) (95% CI) | | [17, 18, 34] |
| Deciding to be comfort measures only (CMO) if *Full Code* after failed NIMV | 0.18 | | 0.01 - 0.53 | Published estimates (Median, Range) | | [23, 29, 30] |
| Deciding to be comfort measures only (CMO) if *DNI* after failed NIMV | 0.98 | | 0.90 - 1.00 | Expert Opinion | |  |
| Surviving CMO | 0.00 | | 0.00 - 0.20 (0.00 - 1.00)* | Random Effects Pooling (2 studies) (95% CI) | | [21, 35] |
| Surviving no mechanical ventilation if moderately ill | 0.86 | | 0.78-0.91  (0.00 - 1.00)* | Single study (95%CI) | | [24] |
| Surviving admission to a regular ward if severe respiratory exacerbation | 0.30 | | 0.00 - 0.64 | Back calculation | |  |
| Discharge to Extended Care Facility after complicated ETT | 0.76 | | 0.55 - 0.91 | Single study (95%CI) | | [5] |
| Discharge to Extended Care Facility after uncomplicated ETT | 0.30 | | 0.22 -0.66 | Single study (95%CI) | | [5] |
| Discharge to Extended Care Facility after NIMV | 0.05 | | 0.01 - 0.10 | Expert opinion | |  |
| Discharge to Extended Care Facility after no NIMV (no mechanical ventilation used) | 0.05 | | 0.05 - 0.08 | Expert opinion | |  |
| Discharge to Extended Care Facility after CMO | 1.00 | | 0.80 - 1.00 (0.00 - 1.00)* | Expert opinion | |  |
| **Life Expectancy** | | | | | | |
| Life expectancy for Mild COPD | 26.60 | 15.00 - 27.00† (0.10 - 30.00)* | | | Single study | [36] |
| Life expectancy for Moderate COPD | 10.00 | 5.00 - 15.00†  (0.10 - 30.00)* | | | Single study | [36] |
| Life expectancy for Severe COPD | 2.70 | 0.50 - 5.40 † (0.10 - 30.00)* | | | Single study | [36] |
| Life expectancy if Extended Care Facility (assumed to be the same for all COPD) | 1.80 | 1.00 - 5.00 † (0.10 - 10.00) * | | | Single study (95%CI) | [37] |
| Life expectancy if Extended Care Facility for short term stay **‡** | Mild COPD  26.60 | 15.00 - 27.00 † (0.10 - 30.00) * | | | Expert Opinion |  |
|  | Moderate COPD  10.00 | 5.00 - 15.00 † (0.10 - 30.00) * | | |  |  |
|  | Severe COPD  2.70 | 0.50 - 5.40 † (0.10 - 30.00)* | | |  |  |
| **Utilities‡** | | | | | | |
| Utility of being at Home (baseline utility) | Mild COPD  0.80 | 0.60 - 1.00 (0.00 - 1.00) * | | | Single study  (95%CI) | [38] |
|  | Moderate COPD  0.73 | 0.50 - 1.00 (0.00 - 1.00) * | | |  |  |
|  | Severe COPD  0.65 | 0.30 - 0.80 (0.00 - 1.00) * | | |  |  |
| Utility of being in Extended Care Facility for long-term | Mild COPD  0.60 | 0.40 - 0.80 (0.00 - 1.00) * | | | Single study (95%CI) | [38] |
|  | Moderate COPD  0.55 | 0.45 - 0.65 (0.00 - 1.00) * | | |  |  |
|  | Severe COPD  0.30 | 0.20 - 0.50 (0.00 - 1.00) * | | |  |  |
| Utility of being in Extended Care Facility for short term stay § | Mild COPD  0.73 | 0.60 - 0.80 (0.00 - 1.00) * | | | Expert Opinion |  |
|  | Moderate COPD  0.67 | 0.55 - 0.73 (0.00 - 1.00) * | | |  |  |
|  | Severe COPD  0.60 | 0.30 - 0.65 (0.00 - 1.00) * | | |  |  |

**Table 1: Parameter Estimates and Data Sources**

*used extreme values for sensitivity analysis for threshold determination and to ask “*what if ?*” questions.

**†**used *LE adjuster* variable to ensure that all life expectancies were varied in parallel when life expectancy was changed. ( range used for sensitivity analysis was 0.56 – 2.78)

**‡** LE in short-term ECF was assumed to be the same as baseline LE before hospitalization.

**§**The utility of discharge to a short term ECF was higher than for discharge to ECF but lower than discharge to home and was derived by averaging the utilities of home and long-term ECF.

║The utility of long-term ECF and of ETT complications were varied in sensitivity analyses using the range of utilities generated from the hypothetical time tradeoff scenarios as described in the text.

¶ For baseline analyses we did not enter a utility for complications of ETT.

References

1. Sin DD, Golmohammadi K, Jacobs P: **Cost-effectiveness of inhaled corticosteroids for chronic obstructive pulmonary disease according to disease severity.** Am J Med 2004, **116**(5):325-331.

2. Donaldson GC, Seemungal TA, Bhowmik A, Wedzicha JA: **Relationship between exacerbation frequency and lung function decline in chronic obstructive pulmonary disease.** Thorax 2002, **57**(10):847-852.

3. Seemungal TA, Donaldson GC, Paul EA, Bestall JC, Jeffries DJ, Wedzicha JA: **Effect of exacerbation on quality of life in patients with chronic obstructive pulmonary disease.** Am J Respir Crit Care Med 1998, **157**(5 Pt 1):1418-1422.

4. Cydulka RK, McFadden ER,Jr, Emerman CL, Sivinski LD, Pisanelli W, Rimm AA: **Patterns of hospitalization in elderly patients with asthma and chronic obstructive pulmonary disease.** American Journal of Respiratory & Critical Care Medicine 1997, **156**(6):1807-1812.

5. Nevins ML, Epstein SK: **Predictors of outcome for patients with COPD requiring invasive mechanical ventilation.** Chest 2001, **119**(6):1840-1849.

6. Plant PK, Owen JL, Elliott MW: **Early use of non-invasive ventilation for acute exacerbations of chronic obstructive pulmonary disease on general respiratory wards: a multicentre randomised controlled trial.** Lancet 2000, **355**(9219):1931-1935.

7. Brochard L, Mancebo J, Wysocki M, Lofaso F, Conti G, Rauss A, Simonneau G, Benito S, Gasparetto A, Lemaire F: **Noninvasive ventilation for acute exacerbations of chronic obstructive pulmonary disease.** N Engl J Med 1995, **333**(13):817-822.

8. Dikensoy O, Ikidag B, Filiz A, Bayram N: **Comparison of non-invasive ventilation and standard medical therapy in acute hypercapnic respiratory failure: a randomised controlled study at a tertiary health centre in SE Turkey.** Int J Clin Pract 2002, **56**(2):85-88.

9. Hoo GW, Hakimian N, Santiago SM: **Hypercapnic respiratory failure in COPD patients: response to therapy.** Chest 2000, **117**(1):169-177.

10. Torrance GW, Boyle MH, Horwood SP: **Application of multi-attribute utility theory to measure social preferences for health states.** Oper Res 1982, **30**(6):1043-1069.

11. Soler-Cataluna JJ, Martinez-Garcia MA, Roman Sanchez P, Salcedo E, Navarro M, Ochando R: **Severe acute exacerbations and mortality in patients with chronic obstructive pulmonary disease.** Thorax 2005, **60**(11):925-931.

12. Bott J, Carroll MP, Conway JH, Keilty SE, Ward EM, Brown AM, Paul EA, Elliott MW, Godfrey RC, Wedzicha JA: **Randomised controlled trial of nasal ventilation in acute ventilatory failure due to chronic obstructive airways disease.** Lancet 1993, **341**(8860):1555-1557.

13. Esteban A, Anzueto A, Frutos F, Alia I, Brochard L, Stewart TE, Benito S, Epstein SK, Apezteguia C, Nightingale P, Arroliga AC, Tobin MJ, Mechanical Ventilation International Study G: **Characteristics and outcomes in adult patients receiving mechanical ventilation: a 28-day international study.** JAMA 2002, **287**(3):345-355.

14. Esteban A, Ferguson ND, Meade MO, Frutos-Vivar F, Apezteguia C, Brochard L, Raymondos K, Nin N, Hurtado J, Tomicic V, Gonzalez M, Elizalde J, Nightingale P, Abroug F, Pelosi P, Arabi Y, Moreno R, Jibaja M, D'Empaire G, Sandi F, Matamis D, Montanez AM, Anzueto A, VENTILA Group: **Evolution of mechanical ventilation in response to clinical research.** Am J Respir Crit Care Med 2008, **177**(2):170-177.

15. Scala R, Nava S, Conti G, Antonelli M, Naldi M, Archinucci I, Coniglio G, Hill NS: **Noninvasive versus conventional ventilation to treat hypercapnic encephalopathy in chronic obstructive pulmonary disease.** Intensive Care Med 2007, **33**(12):2101-2108.

16. Rivera-Fernandez R. Navarrete-Navarro P. Fernandez-Mondejar E. Rodriguez-Elvira M. Guerrero-Lopez F. Vazquez-Mata G. Project for the Epidemiological Analysis of Critical Care Patients (PAEEC) Group: **Six-year mortality and quality of life in critically ill patients with chronic obstructive pulmonary disease.** Crit Care Med 2006, **34**(9):2317-2324.

17. Conti G, Antonelli M, Navalesi P, Rocco M, Bufi M, Spadetta G, Meduri GU: **Noninvasive vs. conventional mechanical ventilation in patients with chronic obstructive pulmonary disease after failure of medical treatment in the ward: a randomized trial.** Intensive Care Med 2002, **28**(12):1701-1707.

18. Squadrone E, Frigerio P, Fogliati C, Gregoretti C, Conti G, Antonelli M, Costa R, Baiardi P, Navalesi P: **Noninvasive vs invasive ventilation in COPD patients with severe acute respiratory failure deemed to require ventilatory assistance.** Intensive Care Med 2004, **30**(7):1303-1310.

19. Confalonieri M. Garuti G. Cattaruzza MS. Osborn JF. Antonelli M. Conti G. Kodric M. Resta O. Marchese S. Gregoretti C. Rossi A. Italian noninvasive positive pressure ventilation (NPPV) study group: **A chart of failure risk for noninvasive ventilation in patients with COPD exacerbation.** European Respiratory Journal 2005, **25**(2):348-355.

20. Celikel T, Sungur M, Ceyhan B, Karakurt S: **Comparison of noninvasive positive pressure ventilation with standard medical therapy in hypercapnic acute respiratory failure.** Chest 1998, **114**(6):1636-1642.

21. Scala R, Naldi M, Archinucci I, Coniglio G, Nava S: **Noninvasive positive pressure ventilation in patients with acute exacerbations of COPD and varying levels of consciousness.** Chest 2005, **128**(3):1657-1666.

22. Vitacca M, Clini E, Rubini F, Nava S, Foglio K, Ambrosino N: **Non-invasive mechanical ventilation in severe chronic obstructive lung disease and acute respiratory failure: short- and long-term prognosis.** Intensive Care Med 1996, **22**(2):94-100.

23. Carratu P, Bonfitto P, Dragonieri S, Schettini F, Clemente R, Di Gioia G, Loponte L, Foschino Barbaro MP, Resta O: **Early and late failure of noninvasive ventilation in chronic obstructive pulmonary disease with acute exacerbation.** Eur J Clin Invest 2005, **35**(6):404-409.

24. Plant PK, Owen JL, Elliott MW: **Non-invasive ventilation in acute exacerbations of chronic obstructive pulmonary disease: long term survival and predictors of in-hospital outcome.** Thorax 2001, **56**(9):708-712.

25. Confalonieri M, Parigi P, Scartabellati A, Aiolfi S, Scorsetti S, Nava S, Gandola L: **Noninvasive mechanical ventilation improves the immediate and long-term outcome of COPD patients with acute respiratory failure.** European Respiratory Journal 1996, **9**(3):422-430.

26. Bardi G, Pierotello R, Desideri M, Valdisserri L, Bottai M, Palla A: **Nasal ventilation in COPD exacerbations: early and late results of a prospective, controlled study.** European Respiratory Journal 2000, **15**(1):98-104.

27. Carrera M, Marin JM, Anton A, Chiner E, Alonso ML, Masa JF, Marrades R, Sala E, Carrizo S, Giner J, Gomez-Merino E, Teran J, Disdier C, Agusti AG, Barbe F: **A controlled trial of noninvasive ventilation for chronic obstructive pulmonary disease exacerbations.** J Crit Care 2009, .

28. Esteban A, Frutos F, Tobin MJ, Alia I, Solsona JF, Valverdu I, Fernandez R, de la Cal MA, Benito S, Tomas R: **A comparison of four methods of weaning patients from mechanical ventilation. Spanish Lung Failure Collaborative Group.** N Engl J Med 1995, **332**(6):345-350.

29. Chu CM, Chan VL, Wong IW, Leung WS, Lin AW, Cheung KF: **Noninvasive ventilation in patients with acute hypercapnic exacerbation of chronic obstructive pulmonary disease who refused endotracheal intubation.** Crit Care Med 2004, **32**(2):372-377.

30. Chu CM, Chan VL, Lin AW, Wong IW, Leung WS, Lai CK: **Readmission rates and life threatening events in COPD survivors treated with non-invasive ventilation for acute hypercapnic respiratory failure.** Thorax 2004, **59**(12):1020-1025.

31. Kramer N, Meyer TJ, Meharg J, Cece RD, Hill NS: **Randomized, prospective trial of noninvasive positive pressure ventilation in acute respiratory failure.** American Journal of Respiratory & Critical Care Medicine 1995, **151**(6):1799-1806.

32. Phua J, Kong K, Lee KH, Shen L, Lim TK: **Noninvasive ventilation in hypercapnic acute respiratory failure due to chronic obstructive pulmonary disease vs. other conditions: effectiveness and predictors of failure.** Intensive Care Med 2005, **31**(4):533-539.

33. Paus-Jenssen ES, Reid JK, Cockcroft DW, Laframboise K, Ward HA: **The use of noninvasive ventilation in acute respiratory failure at a tertiary care center.** Chest 2004, **126**(1):165-172.

34. Ely EW, Baker AM, Evans GW, Haponik EF: **The distribution of costs of care in mechanically ventilated patients with chronic obstructive pulmonary disease.** Crit Care Med 2000, **28**(2):408-413.

35. Crummy F, Buchan C, Miller B, Toghill J, Naughton MT: **The use of noninvasive mechanical ventilation in COPD with severe hypercapnic acidosis.** Respir Med 2007, **101**(1):53-61.

36. Celli BR, Cote CG, Marin JM, Casanova C, Montes de Oca M, Mendez RA, Pinto Plata V, Cabral HJ: **The body-mass index, airflow obstruction, dyspnea, and exercise capacity index in chronic obstructive pulmonary disease.** N Engl J Med 2004, **350**(10):1005-1012.

37. van Dijk PT, Mehr DR, Ooms ME, Madsen R, Petroski G, Frijters DH, Pot AM, Ribbe MW: **Comorbidity and 1-year mortality risks in nursing home residents.** J Am Geriatr Soc 2005, **53**(4):660-665.

38. Tengs TO, Wallace A: **One thousand health-related quality-of-life estimates.** Med Care 2000, **38**(6):583-637.
